# Bovine Serum Albumin Nanoparticles Prepared with Genipin: Size Modulation, Encapsulation, and In Vitro Response

**DOI:** 10.3390/nano16171086

**Published:** 2026-08-31

**Authors:** Beatriz Teixeira, Mónica Almeida, Carolina Frazão, Ana Violeta Girão, Adriana C. S. Pais, Sónia A. O. Santos, Miguel Oliveira, Ricardo J. B. Pinto

**Affiliations:** 1 CESAM-Center for Environmental and Marine Studies, Department of Biology, University of Aveiro, 3810-193 Aveiro, Portugal; beatrizteixeira99@ua.pt (B.T.); monica.alm@ua.pt (M.A.); carolina.frazao@ua.pt (C.F.); a.c.p.s@ua.pt (A.C.S.P.); 2 CICECO-Aveiro Institute of Materials, Department of Materials and Ceramic Engineering, University of Aveiro, 3810-193 Aveiro, Portugal; avgirao@ua.pt; 3 CICECO-Aveiro Institute of Materials, Department of Chemistry, University of Aveiro, 3810-193 Aveiro, Portugal; santos.sonia@ua.pt; 4 CERES-Chemical Engineering and Renewable Resources for Sustainability, Department of Chemical Engineering, University of Coimbra, Pólo II, R. Sílvio Lima, 3030-790 Coimbra, Portugal

**Keywords:** bovine serum albumin, genipin, protein-based nanoparticles, nanocarriers, cancer drug delivery

## Abstract

Protein-based nanoparticles (NPs) are attracting increasing attention as sustainable platforms for advanced biomedical applications. However, achieving high encapsulation efficiencies without compromising nanoparticle stability and biocompatibility remains a key challenge for these bio-based delivery systems. In this context, bovine serum albumin (BSA) NPs crosslinked with genipin were developed as green and biocompatible nanocarriers with tunable physicochemical properties. The synthesis conditions were systematically optimized by varying reaction time, temperature, and the BSA:genipin ratio, with 24 h at room temperature and a 10:1 ratio yielding the most suitable NPs in terms of formation and colloidal stability. The optimized BSA NPs were then used to encapsulate sertraline and 5-fluorouracil, achieving encapsulation efficiencies of 82% and 40%, respectively. These results highlight the ability of this nanoplatform to incorporate bioactive compounds with distinct physicochemical characteristics, with the sertraline-loaded NPs showing particularly high loading efficiency for an albumin-based system. In vitro assays performed on normal prostate epithelial cells (PNT-2) and prostate cancer cells (22Rv1) showed that the BSA NPs modulated drug-induced cytotoxicity relative to free drugs, indicating a formulation-dependent biological response. These findings highlight the potential of genipin-crosslinked BSA NPs as a versatile and sustainable platform for drug delivery in cancer therapy.

## 1. Introduction

Nanotechnology has enabled transformative advances in various sectors, with medicine emerging as a particularly promising field [1,2]. In nanomedicine, nanoscale materials are being used to improve disease diagnosis, treatment, and prevention, especially through the development of advanced drug delivery systems that enhance therapeutic efficacy while minimizing side effects [3,4].

Nanoparticles (NPs), in particular, have shown great potential in optimizing existing pharmaceuticals by improving their stability and bioavailability [1]. This is especially relevant for diseases such as cancer, characterized by uncontrolled abnormal cell growth, that require improved efficiency for diagnostic and therapeutic strategies [5] and could greatly benefit from their potential. Despite the availability of multiple treatment options (e.g., radiotherapy, chemotherapy, surgery, and more recently, hormone therapy), these methods often cause severe and irreversible side effects, compromising the patient’s health and quality of life [6]. Additionally, conventional cancer treatments are costly, non-specific, and prone to resistance development by neoplastic cells [4,7]. As a result, there is a pressing need for novel, cost-effective therapies that precisely target cancer cells while minimizing damage to healthy tissues [1,8].

NPs offer a promising solution to address the aggressive nature of cancer cells and mitigate the complications caused by current treatments [1,3]. Among the various types, the organic variants, particularly polymeric and protein-based NPs, have garnered significant attention for drug delivery applications [9,10].

Protein-based NPs have been increasingly studied for biomedical applications due to their biocompatibility, bioactivity, non-immunogenicity, structural versatility, and ability to incorporate and deliver a wide range of therapeutic compounds [11,12,13]. Recent advances in nanocarrier design have further emphasized the importance of tailoring physicochemical properties and developing stimuli-responsive strategies to overcome biological barriers and improve tumour penetration, cellular uptake, and targeted drug delivery [14]. Among these nanocarriers, bovine serum albumin (BSA) and human serum albumin (HSA) are particularly promising for cancer therapy due to their biocompatibility, stability, and ability to bind a wide range of drugs. While BSA is widely used due to its availability and corresponding cost-effectiveness, HSA is valued for its compatibility with human physiology, making both suitable for enhancing drug solubility, targeted delivery, and controlled release [15]. BSA was selected for this study due to its widespread use in preclinical studies, affordability, and suitability for large-scale NP production, which makes it an ideal candidate for drug delivery applications and in vitro diagnostic studies [16]. Conventional crosslinkers like glutaraldehyde are effective in BSA NPs preparation but pose significant toxicity concerns, limiting their use in biomedical applications. Genipin, a natural compound derived from *Gardenia jasminoides*, offers a safer alternative. It enables efficient crosslinking of amine-containing polymers while significantly improving biocompatibility, making it highly suitable for medical and pharmaceutical use [17,18].

Prostate cancer (PCa) is one of the most prevalent types of cancer in men worldwide [5]. Given the urgent need for more effective treatments, several drugs originally developed for non-cancer indications, including cardiovascular, antimicrobial, anti-inflammatory, and psychiatric medications, have been investigated for their potential in PCa therapy [19], supported by advances in genomic and proteomic technologies in recent years. Among the most promising approaches is drug repurposing, which offers a faster and more cost-effective alternative to traditional drug development by leveraging already approved compounds and bypassing early clinical trial stages [20]. In this study, sertraline (STR, C_17_H_17_C_l2_N) and 5-fluorouracil (5-FU, C_4_H_3_FN_2_O_2_) were selected based on their demonstrated potential in cancer research. STR, an antidepressant, has shown cytotoxic effects against various cancer cell lines [21,22,23], while 5-FU is an established chemotherapy drug widely used in cancer treatment [24]. However, both drugs present significant limitations. STR has been associated with cytotoxicity in normal cells, while 5-FU faces increasing challenges due to cancer cell resistance [25]. Encapsulating these drugs in NPs may help mitigate these issues by allowing better control over the dosage of 5-FU or STR.

The integration of nanomedicine with drug repurposing (using already approved pharmaceuticals) offers a cost-effective and efficient approach to improve cancer treatment, reducing drug development time and benefiting patients [26,27]. However, further research is required to overcome challenges, such as adverse side effects, systemic toxicity, and resistance development in cancer cell lines.

Thus, this study focuses on a key challenge in the development of novel protein-based nanocarriers by introducing a greener and safer strategy for the encapsulation of STR and 5-FU into BSA NPs, using genipin as a natural and non-toxic cross-linker instead of conventional agents like glutaraldehyde. The synthesis process was systematically optimized by fine-tuning reaction time, temperature, and the BSA:genipin ratio to obtain stable, well-defined NPs with enhanced drug loading efficiency. Importantly, the biological response of the optimized NPs was assessed in both normal (PNT-2) and prostate cancer (22Rv1) cell lines, providing relevant insight into their safety profile and early-stage prostate cancer therapy potential.

## 2. Materials and Methods

### 2.1. Chemicals

Genipin (CAS 6902-77-8) was purchased from Challenge Bioproducts Co., Ltd. (Douliu City, Taiwan, China); sertraline (STR) hydrochloride (CAS 79559-97-0), 5-fluorouracil (5-FU) (CAS 51-21-8) and 3-(4,5-dimethyl-2-thiazolyl)-2,5-diphenyltetrazolium bromide (MTT) (CAS 298-93-1) were purchased from Tokyo Chemical Industry (TCI Chemicals, Zwijndrecht, Belgium). Bovine serum albumin (BSA) (CAS 9048-46-8) and the remaining reagents (all analytical grade) were purchased from Sigma-Aldrich (Madrid, Spain).

Stock solutions of STR (27.3 mg/mL) and genipin (780 mg/mL) were prepared in dimethyl sulfoxide (DMSO, Fisher Scientific, Loughborough, UK), whereas the stock solution of 5-FU (1.5 mg/mL) was prepared in ultrapure water. To attain the desired working concentrations for the in vitro cell viability assays, the stock solutions were diluted in RPMI-1640 medium, and sterilization was achieved by filtration through a 0.22 µm pore-size polyethersulfone filter.

### 2.2. Synthesis of BSA and Encapsulation of STR and 5-FU

The synthesis of BSA NPs and the encapsulation of the active components, STR and 5-FU, was carried out generally following the methodology developed by Luo et al. (2020) [28], testing different variables, such as reaction duration (24 h and 72 h), temperature of reaction (room temperature and 37 °C) and different ratios of BSA and genipin (10:4, 10:1, 20:1, 40:1). Thus, BSA (400 mg) was dissolved in 20 mL of ultrapure water and genipin was used as a crosslinker (40 mg of genipin dissolved in 2 mL of acetone. The genipin solution was added dropwise to the BSA solution to initiate the reaction. The solution was sonicated (Vibracel Sonifier, Sonics VCX 500, Sonics & Materials, Newtown, CT, USA) for 10 min at 40% amplitude to accelerate the reaction. This suspension was prepared in triplicate.

For the encapsulation procedure, 2.5 mg of the active compounds STR or 5-FU were dissolved in 2 mL of ultrapure water. Each drug solution was then added to the BSA/genipin suspension, and the resulting mixture was maintained under magnetic stirring for 24 h to allow drug encapsulation. All the suspensions were then subjected to three consecutive washing steps by centrifugation (24,652× *g* for 15 min at 4 °C). After each centrifugation step, the supernatant was collected, and the pellet was resuspended in ultrapure water and the NPs redispersed using probe sonication for 10 min at 40% amplitude. Following the final washing step, the NPs pellet was resuspended in ultrapure water and stored at 4 °C until further characterization and use.

### 2.3. Determination of Encapsulation Efficiency of STR and 5-FU

Ultra-high-performance liquid chromatography (UHPLC) was used to determine the encapsulation efficiency (EE%) of the BSA NPs with STR (BSA_STR) and BSA NPs with 5-FU (BSA_5-FU). Samples were filtered using nylon filters with a pore diameter of 0.45 µm for UHPLC-UV analysis. Aliquots of 15 µL were injected into the UHPLC system, which consisted of an Ultimate 3000 pump, an Ultimate 3000 autosampler (set to 16 °C), and an Ultimate 3000 photodiode array detector (DAD) (Thermo Fisher Scientific, San Jose, CA, USA). The chromatographic separation was performed on a Hypersil Gold RP C18 column (100 × 2.1 mm, 1.9 µm particle size) preceded by a C_18_ pre-column (10 × 2.1 mm, 1.9 µm particle size), both from Thermo Fisher Scientific, and maintained at 40 °C. The mobile phase was composed of (A) water (99:1, *v*/*v*) and (B) acetonitrile, both containing 0.1% (*v*/*v*) formic acid. A gradient elution was used at a flow rate of 0.35 mL min^−1^ for 24 min as follows: 1–42% B from 0 to 8 min; 42–100% B from 8 to 14 min; 100% B from 14 to 15 min; 100–1% B from 15 to 20 min, and kept at 100% B from 20 to 24 min. Chromatograms were recorded at 225 nm. Data acquisition was managed through the Xcalibur^®^data system (Thermo Finnigan, San Jose, CA, USA). For the calibration curve, standard solutions of 1, 2.5, 5, 10, 25, 50, and 102 mg/mL of STR and 5-FU were prepared by dilution in ultrapure water. The linearity of the method was established, and the regression equations were found: *y* = 2 × 10^8^ *x* + 144,432 (*r*^2^ = 0.9997) and *y* = 1 × 10^8^ *x* + 640,011 (*r*^2^ = 0.9966), for STR and 5-FU, respectively, where *y* is the peak area and *x* is the concentration in µg/mL. The limit of detection (2.4 µg/mL for STR and 13.0 µg/mL for 5-FU) and limit of quantitation (7.4 µg/mL for STR and 39.4 µg/mL for 5-FU) were determined as three and ten times the noise level (S/N), respectively. Following the active ingredient concentration determination, the EE% of STR and 5-FU was calculated using the following equation:

EE% = [mass(compound)_initial_ − mass(compound)_supernatant_/mass(compound)_initial_] × 100

### 2.4. Characterization of the Synthesized NPs

Dynamic Light Scattering (DLS) was used to characterize the hydrodynamic size and the polydispersity index (PDI) of the NPs, and Electrophoretic Light Scattering (ELS) to determine the zeta potential, which provides information on the stability of empty and compound-loaded BSA NPs (Malvern ZetaSizer Nano-ZS, Malvern Panalytical, Oeiras, Portugal). The analysis settings considered room temperature, samples dispersed in water (refractive index 1.330), and the utilization of “Polystyrene latex” as the material (refractive index 1.590 and absorption 0.010). Each sample was diluted 1:10 in ultrapure water and underwent three measurements, and the data are presented as the mean ± standard deviation, with the hydrodynamic size expressed as the Z-average size. Scanning transmission electron microscopy (STEM) was used to characterize the particles in terms of morphology and size. STEM images were captured using a scanning electron microscope (SEM) Hitachi SU-70, equipped with a field emission gun (FEG), (Hitachi High-Technologies Corporation, Tokyo, Japan) operating at a 4 kV or 15 kV accelerating voltage. The sample preparation involved placing a droplet of the sample directly onto a carbon grid, allowing the solvent to evaporate.

### 2.5. Biological Effects of BSA NPs

#### 2.5.1. Biological Models

Two cell lines were used in this study: the PNT-2 cell line, representing normal prostate cells (generously provided by Dr. Paula Guedes Pinto of the Faculty of Pharmacy, Porto University, Portugal), and the 22Rv1 cell line (CRL-2505, kindly provided by Dr. João Ramalho from the Netherlands Cancer Institute in Amsterdam, the Netherlands), representative of PCa cells. Both cell lines were cultured in RPMI-1640 medium supplemented with 2 mM L-glutamine (Biowest, Nuaillé, France), 10% fetal bovine serum (FBS) (Capricorn Scientific, Ebsdorfergrund, Germany), 100 μg/mL streptomycin (Capricorn Scientific, Ebsdorfergrund, Germany), 100 U/mL penicillin G (Capricorn Scientific, Ebsdorfergrund, Germany), and 2.5 μg/mL amphotericin B (Biowest, Nuaillé, France). Routine maintenance was performed in 10 cm diameter cell culture dishes, with cells incubated at 37 °C in a humidified incubator with 5% carbon dioxide.

#### 2.5.2. Experimental Design

##### Individual Exposure

Cells were seeded at a density of 10,000 cells per well in 96-well plates. After overnight adhesion, the cells were exposed to various conditions: genipin (used in the BSA NPs synthesis), STR and 5-FU (active drugs encapsulated in the BSA NPs), BSA NPs (without drug), BSA_STR (NPs with STR), and BSA_5-FU (NPs with 5-FU). Concentrations tested ranged from 0.313 to 20 mg/L for genipin; 0.003 to 50 mg/L for STR, 0.078 to 15 mg/L for 5-FU, 1.563 to 100 mg/L for BSA NPs (without drug), 0.016 to 250 mg/L for BSA_STR, and 0.391 to 50 mg/L for BSA_5-FU. Cell viability was evaluated at three different time points, 24, 48, and 72 h.

##### Cell Viability Assessment: 3-[4,5-Dimethylthiazol-2-yl]-2,5-Diphenyltetrazolium Bromide (MTT) Assay

The MTT assay was used to assess cell viability through cells’ metabolic activity quantification, based on the conversion of the yellow tetrazolium salt solution to purple formazan crystals. Thus, after exposure for 24, 48, and 72 h to the different experimental conditions, the cells were washed with phosphate-buffered saline (PBS) solution, and MTT (0.5 mg/mL) was added. Following a two-hour incubation period, the formed formazan crystals were solubilized with DMSO, and absorbance was measured at 570 nm (maximum formazan absorbance) and 690 nm (baseline) using a microplate reader (Multiskan Spectrum, Thermo Scientific, Porto Salvo, Portugal). Viability was expressed as a percentage of control.

### 2.6. Data Analysis

Inhibitory concentrations (IC_50_) were calculated through nonlinear regression analysis with variable slope (four parameters) using GraphPad Prism software (version 8.02). To assess the impact of the tested concentrations on the two cell lines, at designated time points (24, 48, and 72 h), a paired *t*-test was conducted. Statistical significance was set at *p* < 0.05. Additionally, a two-way analysis of variance (ANOVA) was employed to explore the potential interaction of exposure periods and tested concentrations. Post-hoc analysis was performed using the Tukey test, and the significance level was set at *p* < 0.05 (* denotes significant differences between concentrations per time point, and # denotes significant differences to the 24 h time point).

## 3. Results and Discussion

### 3.1. Synthesis and Characterization of BSA NPs and Encapsulation of STR and 5-FU

The synthesis procedure investigated the effects of reaction duration, temperature, and the ratio of BSA and genipin on NPs properties to achieve improved characteristics (e.g., size, PDI, and zeta potential) for encapsulation of active components (STR and 5-FU). The physicochemical features of the resulting particles were analyzed by DLS and ELS and are summarized in Table 1.

The first parameter assessed for synthesis optimization was reaction time, at 24 and 72 h. Shahnholian et al. (2017) reported that BSA NPs synthesis using genipin takes 24 h [29], which is consistent with the data from the present study. After 24 h, a bluish colloidal suspension was formed, and with a higher reaction time (72 h), the suspension gradually became more gelatinous. This aggregation was accompanied by a shift in zeta potential from −11 mV to +4.4 mV and an increase in PDI from 0.151 to 1 after 24 and 72 h, respectively (Table 1), indicating significant particle destabilization. In addition to 24 h and 72 h, intermediate reaction times of 8 h and 48 h were also visually assessed to better understand NPs formation. At 8 h, almost no NPs formation was observed, suggesting that this reaction time was insufficient for efficient NP formation under the conditions tested. At 48 h, signs of significant aggregation were already noticeable, similar to the trend observed at 72 h. Liu et al. (2023), who tested 24 and 48 h, also reported that 24 h was the optimal time for obtaining NPs using genipin as the crosslinking agent [30]. Notably, their study directly evaluated the residual amino group content, which remained stable after 24 h, thereby providing evidence regarding the progression of the crosslinking reaction. However, their selection did not consider the physicochemical properties of the resulting particles. Thus, a 24 h reaction time was selected for the encapsulation process.

The second parameter studied was the temperature of the reaction, as it can influence NP formation and the final properties of BSA NPs. As shown in Table 1, the two temperatures tested (room temperature and 37 °C) yielded markedly different particle sizes. At 37 °C, micrometric particles (>1000 nm) were formed, whereas at room temperature, nanometric particles (292 nm) were obtained. The increase in particle size may be related to the inherent tendency of globular proteins like albumin to aggregate at higher temperatures. In addition, previous studies have reported that temperature can influence the kinetics of genipin-mediated crosslinking [18,31], which may also contribute to the differences in particle formation observed here. However, as the degree of crosslinking was not directly quantified in the present study, its contribution to the observed increase in particle size cannot be established. NPs synthesized at the highest temperature also exhibited reduced stability (zeta potential of −4.1 versus −13.5 mV at room temperature). Thus, room temperature was selected for the encapsulation process.

The final parameter assessed in the synthesis procedure was the BSA:genipin ratio, with four different ratios tested: 10:4, 10:1, 20:1, and 40:1. As shown in Table 2, BSA NPs synthesized with a 10:4 ratio exhibited the largest hydrodynamic size (427 nm), likely associated with the lower availability of free amino groups when a higher concentration of crosslinker is used [30], resulting in the formation of larger and more crosslinked structures. For the other ratios, the obtained sizes ranged between 250 and 350 nm, with the ratios 10:1 and 20:1 eliciting particles with smaller sizes and more monodisperse suspensions (PDI values of 0.183 and 0.156, respectively). In terms of zeta potential, all ratios led to values between −27.4 and −20.6 mV, indicating moderate stability. Considering that the 10:1 and 20:1 presented very similar values, the 10:1 ratio was selected for the encapsulation formulation, allowing savings in terms of protein consumption. Overall, considering all the tested variables, the most favorable conditions for synthesizing BSA NPs using genipin involved conducting the desolvation-based reaction at room temperature for 24 h and utilizing a 10:1 (*w*/*w*) BSA:genipin ratio. In addition to the parameters evaluated in this study, the influence of pH should be considered in future optimization, as it may affect BSA properties and its interaction with genipin during NPs formation.

To further characterize the resulting nanoparticles, FTIR analysis of BSA and BSA NPs was performed. The characteristic amide bands of BSA were preserved in the BSA NPs, with only minor shifts in their positions (Figure S1), suggesting that the main structural features of BSA were maintained during the NPs preparation. However, no specific spectral changes could be clearly attributed to genipin-mediated crosslinking.

Considering the obtained data, the next step involved the encapsulation of the STR and 5-FU. The characterization results, presented in Table 2, showed that the unloaded BSA NPs presented a size of 296 nm and may be considered monodisperse. Shahgholian et al. (2017), who also used genipin as a crosslinker to synthesize BSA NPs, obtained a hydrodynamic size range between 153–184.4 nm [29]. Similar results were found in other studies, with sizes ranging between 100 and 200 nm [29,32,33]. However, when active compounds were encapsulated, the hydrodynamic size and PDI decreased to 217 nm and 0.147 for BSA_5-FU but increased to 422 nm and 0.206 for BSA_STR. All formulations exhibited negative zeta potentials ranging from −20 to −28 mV, suggesting some contribution of electrostatic repulsion to NP stability. Shahgholian et al. (2017) also observed a shift in the hydrodynamic size when encapsulating curcumin, from 90 to 120 nm [29].

**Figure 1:**
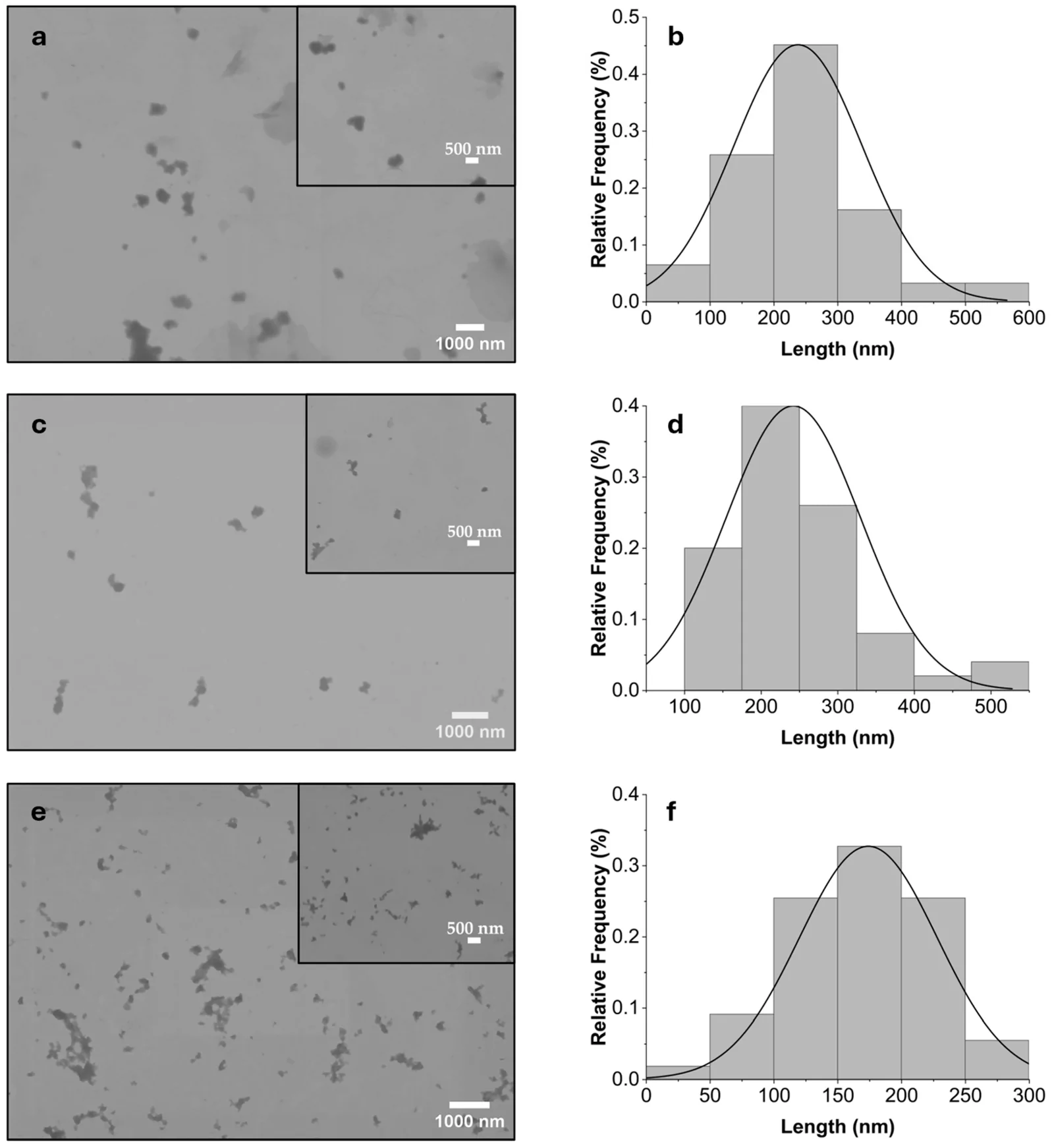
STEM images, including insets with higher magnification, of BSA NPs (**a**) (×7.00 K), BSA_STR (×9.00 K) (**c**), BSA_5-FU (×10.0 K) (**e**), and their size distribution histograms (**b**), (**d**), and (**f**), respectively.

Size is a critical parameter influencing NPs applicability, particularly in biomedical applications, modulating cellular uptake, circulation time, and therapeutic efficacy [30]. However, the morphology of the NPs is also important. The STEM image analysis confirmed that all NPs exhibited a spherical shape (Figure 1a,c,e), with mean sizes of 237 nm for the BSA NPs (Figure 1b), 242 nm for BSA_STR (Figure 1d), and 177 nm for the BSA_5-FU (Figure 1f).

The size differences between unloaded BSA NPs and drug-loaded BSA NPs indicate that STR and 5-FU differently affect the resulting NPs formulations. For BSA_STR, the hydrodynamic diameter increased markedly from 296 to 422 nm, whereas the particle size determined by STEM remained essentially unchanged (237 and 242 nm, respectively). This discrepancy suggests that the increased hydrodynamic diameter may not reflect an increase in the dimensions of individual NPs, but changes occurring in aqueous suspension. In addition to differences in particle hydration, STR loading may promote some degree of particle association, which would increase the apparent hydrodynamic diameter measured by DLS without producing a comparable increase in the individual particle size observed by STEM [34]. The slightly higher PDI of BSA_STR (0.206) compared with unloaded BSA NPs (0.183) is also consistent with a broader particle-size distribution, although these measurements alone cannot confirm aggregation.

In contrast, BSA_5-FU exhibited smaller sizes than unloaded BSA NPs by both DLS (217 vs. 296 nm) and STEM (177 vs. 237 nm), suggesting that 5-FU affects NP formation or organization differently from STR. These contrasting effects may be related to differences in the physicochemical properties of the two drugs and their interactions with BSA during nanoparticle formation. Differences between particle sizes determined by DLS and electron microscopy have also been widely reported for protein-based NPs. For example, Karami et al. (2020) reported DLS-measured NPs sizes of 229 nm and 378 nm, while SEM analysis indicated smaller sizes of 91.77 nm and 97.01 nm, respectively [35]. Similarly, Nosrati et al. (2020) showed that BSA NPs loaded with curcumin had a DLS-measured size of 140 nm, whereas STEM analysis showed a significantly smaller size of 39 nm [33]. These discrepancies can occur because DLS measures the hydrodynamic diameter of particles in suspension and is sensitive to the hydration layer and particle association, whereas electron microscopy measures individual particles in the dry state. Additional factors, including pH, dispersion medium, and differences in the ratio of NPs to the active compound, may also influence the measured particle size. Studies have also shown that changes in BSA concentration and drug loading can influence NPs size [36,37].

### 3.2. Encapsulation Efficiency of the NPs

To quantify the EE%, calibration curves for STR and 5-FU were made using UHPLC. The EE% obtained in this study was approximately 82% for STR and 40% for 5-FU (Table 2), which can be considered high compared with the values already reported in the literature [38,39]. The differences observed between the two drugs can be attributed to their aqueous solubility. 5-FU, being highly water-soluble (12.2 mg/mL), has a lower affinity for the hydrophobic/protein-rich environment of BSA NPs, resulting in reduced encapsulation [40]. In contrast, STR is less water-soluble (3.8 mg/mL) and more lipophilic, which favors its interaction with BSA and entrapment within the NPs matrix [41]. pKa differences may also contribute, since 5-FU (pKa ≈ 8.0) is more ionized at physiological pH than STR (pKa ≈ 9.2), potentially leading to weaker interactions with BSA during nanoprecipitation [42,43]. Other factors such as crystalline state, molecular weight, and drug-polymer interactions may also influence the EE%. It is important to highlight that in all repetitions, the EE% remained consistent, with minimal variation observed between batches.

Regarding the interaction mechanisms, no covalent bonding was designed between the drugs and the BSA NPs. Instead, drug loading is mainly driven by physical entrapment and non-covalent interactions. STR likely interacts with BSA predominantly through hydrophobic forces and hydrogen bonding, whereas 5-FU, being more hydrophilic and ionized, may interact via weaker electrostatic interactions and hydrogen bonds. The EE% of 5-FU in BSA NPs is generally low. For example, Maghsoudi et al. (2008) achieved an EE% of approximately 30% for 5-FU encapsulated in BSA, although glutaraldehyde was used as the crosslinking agent, which may have influenced the results [39]. Yi et al. (1999) also reported values of 31.6 and 33.8% in sodium alginate ^125^I BSA NPs [44]. For STR, Lei et al. (2019) reported an EE% of around 70% using liposomes [45], slightly lower than the value found in the present study. The EE% depends not only on the chemical interaction between components but also on the medium pH and drug properties [36]. Xie et al. (2012) demonstrated that a high density of negative charges on BSA molecules can enhance drug loading by promoting greater dispersion and stronger electrostatic interactions, obtaining an EE% of 99% for doxorubicin encapsulation [36]. Shahgholian et al. (2017) also reported an EE% of 72.54% for curcumin [29]. The high EE% observed in the present study further supports the potential of BSA NPs as a promising system for the delivery of STR and 5-FU.

### 3.3. Biological Effects

**Figure 2:**
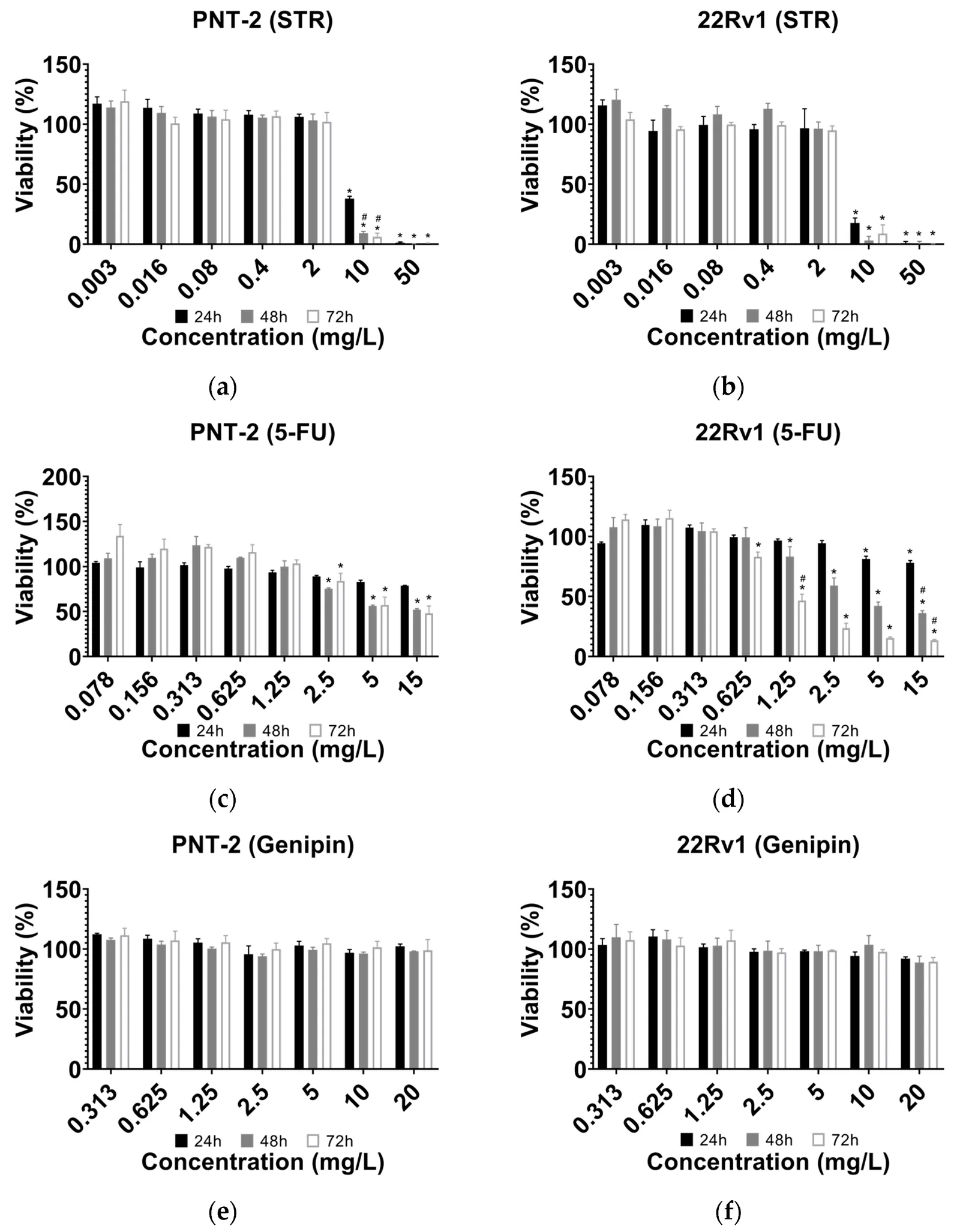
Cell viability of PNT-2 and 22Rv1 cell lines exposed for 24, 48, and 72 h to different concentrations of STR (**a**,**b**), 5-FU (**c**,**d**), and genipin (**e**,**f**). Error bars correspond to the standard error. # denotes significant differences to 24 h time point *p* < 0.05; * denotes significant differences between concentrations per time point, *p* < 0.05.

The assessment of cell viability is an endpoint commonly used to study the impact of xenobiotics in in vitro assays. In this study, the effects of the synthesized NPs (empty or loaded with active substances), the active substances, and chemicals used in the synthesis process were assessed to validate the biocompatibility of the NPs or potential NPs-specific effects. In this sense, the effects of direct exposure to the encapsulated drugs were also tested. STR demonstrated an ability to impact cell viability, exhibiting significant toxicity for PNT-2 (normal) and 22Rv1 (cancerous) cells, especially at the highest concentrations, 10 and 50 mg/L (Figure 2a,b). A paired *t*-test showed significant differences between PNT-2 and 22Rv1 cells at 24 h (*p* = 0.0097). The decreased cell viability allowed estimation of the 72 h IC_50_ of STR for PNT-2 as 4.63 mg/L, and for 22Rv1 as 4.91 mg/L, indicating similar sensitivity of both cell lines (Table 3). Other studies also suggested that STR is a promising drug to fight cancer, leading to cell apoptosis, inhibiting their proliferation, and inducing autophagy in various cancer cell lines [21,46,47,48,49,50]. For instance, He et al. (2024) assessed the effects of STR in human and mouse colorectal cancer cells and revealed that STR suppressed cell growth through activation of autophagic pathways [48,49]. The present study confirms that STR decreases PCa cell viability but exhibits a similar effect on normal prostate cells.

PNT-2 exposure to 5-FU did not induce significant effects on cell viability at 24 h, but decreased cell viability at 48 and 72 h, at 2.5, 5, and 15 mg/L (Figure 2c). For 22Rv1 cells, significant effects were found at concentrations equal to or higher than 5 mg/L. The cytotoxic effects increased over time, with significant effects at 48 h found after exposure to concentrations equal to or higher than 1.25 mg/L, whereas at 72 h, significant effects were found at concentrations equal to or higher than 0.625 mg/L. Significant differences were found between cell lines at 48 h (*p* = 0.0019) and 72 h (*p* = 0.0017). Thus, based on the 72 h IC_50_ of 5-FU for PNT-2 (9.71 mg/L) and for 22Rv1 (1.17 mg/L), it is clear that 5-FU is more toxic to 22Rv1 than PNT-2. The time-dependent effect of 5-FU may be associated with its ability to affect cells in the S phase of the cell cycle and thus becomes noticeable when the cell starts mitosis [24,51], and the 5-FU cytotoxicity varies depending on the cancer cell lines [52,53]. For instance, in the MDA-MB-231 breast cancer cell line, the reported 5-FU 48 h IC_50_ was 68.29 mg/L [52], whereas in the present study, the 48 h IC_50_ for 22Rv1 was 3.43 mg/L. For gastric cancer cell lines MKN-1, MKN-28, MKN-45, and MKN-74, the 72 h IC_50_ values were 0.63 mg/L, 0.19 mg/L, 0.32 mg/L, and 0.34 mg/L, respectively [53]. Although 5-FU is a valuable first-line chemotherapy treatment that reduces the viability of cancer cells, it also affects normal cells, although at a slower rate. Furthermore, the effectiveness of 5-FU treatment can also be compromised by resistance. In this sense, the synthesis of NPs carrying 5-FU may help decrease cell resistance to the compound [54,55]. Encapsulating 5-FU in BSA NPs potentially allows more precise targeting of cancer cells, increasing local 5-FU concentration and overcoming resistance mechanisms such as limited cellular uptake or active drug efflux. Controlled drug release allows extending the therapeutic effect while reducing systemic side effects.

Genipin did not affect the viability of PNT -2 and 22Rv1 cell lines (Figure 2e,f). The absence of significant effects on cell viability prevented the estimation of IC_50_ values. However, significant differences were found between cell lines at 72 h (*p* = 0.0191). This lack of effects on both cell lines supports its use as a green crosslinker for the synthesis of BSA NPs.

**Figure 3:**
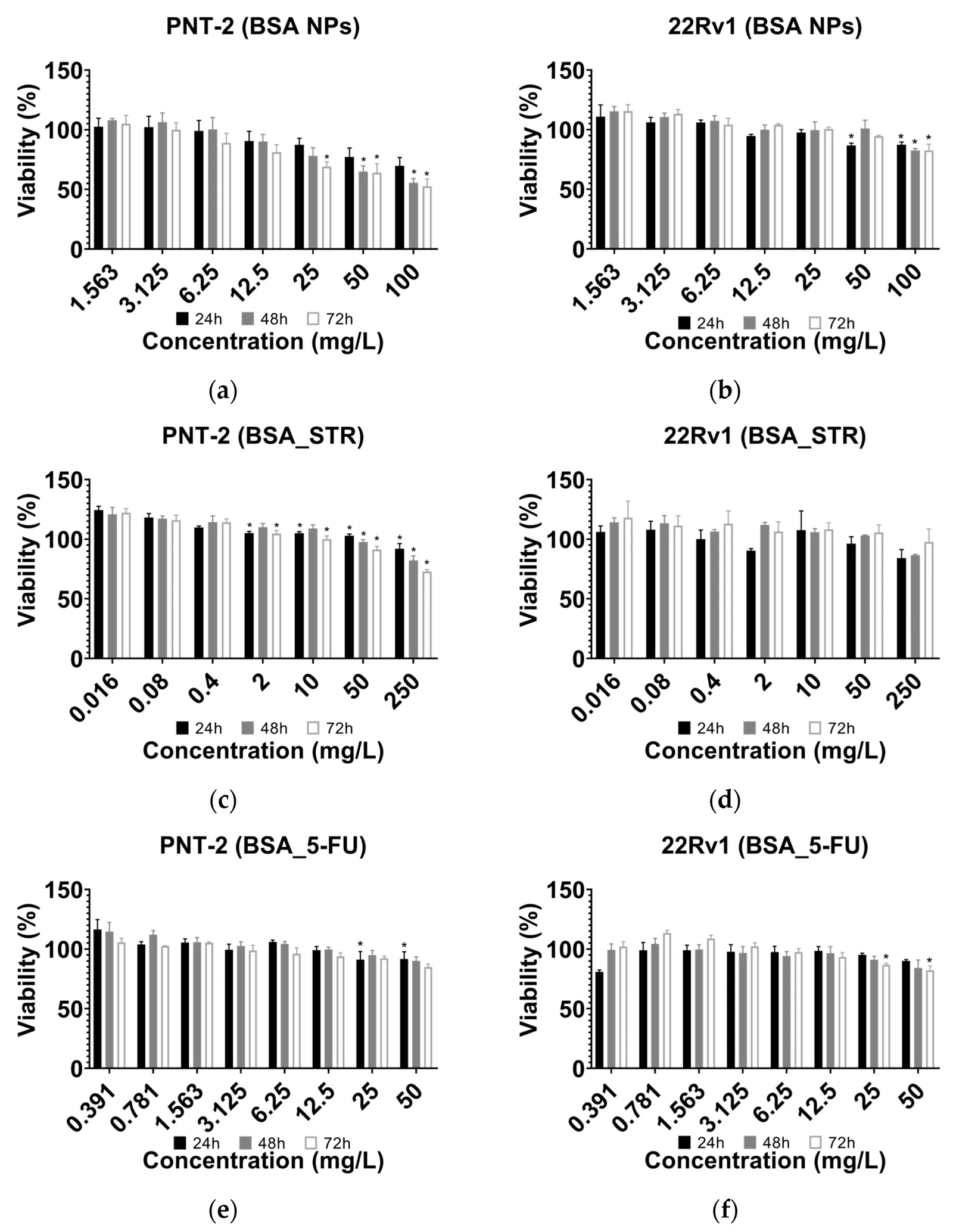
Cell viability of PNT-2 and 22Rv1 cell lines exposed for 24, 48, and 72 h to different concentrations of BSA NPs (**a**,**b**), BSA NPs with encapsulated STR (BSA_STR) (**c**,**d**), and BSA NPs with encapsulated 5-FU (BSA_5-FU) (**e**,**f**). Error bars correspond to the standard error. * Significant differences between concentrations per time point, *p* < 0.05.

BSA NPs demonstrated the ability to affect PNT-2 and 22Rv1 cell viability, with 22Rv1 cells exhibiting lower sensitivity. A time-dependent toxicity increase was found after exposure to BSA NPs (Figure 3a), with significant decreases in PNT-2 cell viability found after 48 h exposure to 50 and 100 mg/L and 72 h exposure to 25, 50, and 100 mg/L. The estimated 48 h and 72 h IC_50_ values for PNT-2 were 205.43 mg/L and 134.45 mg/L. Regarding 22Rv1 cells, as observed for PNT-2 cells, significant viability decreases were observed after 24 h exposure to 50 and 100 mg/L (Figure 3b). However, after 48 and 72 h, only the highest concentration demonstrated cytotoxicity. When comparing the effects of BSA NPs between the two cell lines, significant differences were found at all time points. The 48 and 72 h IC_50_ values for 22Rv1 were 648.21 mg/L and 590.11 mg/L, respectively. The IC_50_ values suggest cell-specific effects that are time-dependent. Overall, the present study data suggest a higher sensitivity of the PNT-2 cell line to BSA NPs than 22Rv1. Gharbavi et al. (2023) demonstrated that BSA NPs (142 nm) alone exhibited effects on glioblastoma cells (U87-MG), but the study did not address the effects on normal cell lines [56]. In other cell lines, such as the breast cancer cell lines MDA-MB-231, MCF-7 [32], and 4T1 [33], as well as the lung cancer cell line A549 [57], BSA NPs (140 nm, synthesized with baicalein, a flavone) did not significantly affect cell viability in the absence of encapsulated drugs. Hsu et al. (2023) demonstrated that BSA NPs (60.74 nm) can exhibit toxicity toward the MCF-7 breast cancer cell line when the particles are synthesized with specific functionalization designed to target cancer cells [32]. BSA NPs used to encapsulate compounds for drug delivery have proven highly effective in delivering chemotherapeutic agents and other drugs, such as doxorubicin and methotrexate. This encapsulation enhances therapeutic efficacy, reduces adverse effects, improves drug selectivity for target cells, and minimizes off-target effects [32,33,56,57].

The amount of encapsulated STR for each tested concentration was calculated to allow a comparison of effects between direct exposure and delivery by NPs. Thus, 0.016, 0.08, 0.4, 2, 10, 50, and 250 mg/L of BSA_STR contain 0.0008, 0.004, 0.02, 0.1, 0.5, 2.5, and 12.5 mg/L of STR, respectively. Cell viability decreased after exposure to BSA_STR NPs concentrations higher than 2 mg/L (Figure 3c). The PNT-2 cells were able to adapt to BSA_STR NPs exposure, with significant effects on cell viability at 48 h only found after exposure to 50 and 250 mg/L. However, at 72 h, the response profile was similar to 24 h. For 22Rv1 cells, despite the slight viability decreases found after exposure to BSA_STR NPs, no significant effects were found up to 72 h (Figure 3d). When comparing the effects of BSA_STR NPs in PNT-2 and 22Rv1 cells, significant differences were found only at 24 h (*p* = 0.0097). The obtained data allowed the estimation of the 72 h IC_50_ of BSA_STR for PNT-2 as 1652.37 mg/L and for 22Rv1 as 4142.35 mg/L (Table 3).

Other studies have reported that encapsulation of biologically active substances in BSA NPs elicits a higher impact on cell viability than the free substance. For example, Keshavarz et al. (2022) reported, following 48 h of exposure, significantly higher cytotoxicity against U-87 MG cells for BSA-Curcumin NPs than free curcumin (IC_50_ values of 17.43 mg/L versus 33.08 mg/L, respectively) [58]. Consistently, Khodear et al. (2024) reported IC_50_ values for HepG2 cells (liver cancer cell line) of 10.3 µg/mL for BSA-berberine NPs and 15.58 µg/mL for free berberine [59]. Ghosh et al. (2022) confirmed that the IC_50_ of BSA-berberine for human glioblastoma LN229 cells after 24 h exposure was 13,600 mg/L, approximately twofold lower than that of free berberine (IC_50_ = 33,400 mg/L), thereby underscoring the superior therapeutic efficacy of BSA-berberine NPS [60].

Cell viability data showed a marked difference between free and encapsulated STR. While 10 mg/L free STR almost completely inhibited cell viability, the same nominal concentration of STR encapsulated within BSA NPs resulted in approximately 50% inhibition. This reduced cytotoxic effect may reflect differences in the cellular availability of STR following encapsulation, potentially associated with its retention within and subsequent release from the NPs. However, as drug release was not directly evaluated in the present study, the contribution of release kinetics to the observed effect cannot be established. Nevertheless, these results are consistent with the findings of Lei et al. (2020) in the Caki-1 renal cell carcinoma cell line, where encapsulation of STR in liposomes reduced its cytotoxicity and provided a drug delivery platform with controlled-release properties [45].

The amount of 5-FU present in the BSA_5-FU NPs was estimated for each tested concentration: 0.390625, 0.78125, 1.5625, 3.125, 6.25, 12.5, 25, and 50 mg/L, which contain 0.1078125, 0.215625, 0.43125, 0.8625, 1.725, 3.45, 6.9, and 13.8 mg/L of 5-FU, respectively. BSA_5-FU had a lower impact on PNT-2 cell viability than BSA_STR (Figure 3c,e). A comparison between the effects of 5-FU alone and encapsulated shows a lower impact of 5-FU when encapsulated. Significant effects on PNT-2 viability by 5-FU encapsulated were only found after 24 h at 25 and 50 mg/L, whereas for 22Rv1 effects were only found after 72 h exposure to the same concentrations of particles. Significant differences in the viability of the two cell lines were found after 48 h exposure to BSA_5-FU. Maghsoudi et al. (2008) demonstrated that 5-FU encapsulated in BSA NPs (210 nm) exhibits the ability to achieve sustained drug release without exhibiting any burst effect [39], demonstrating that sustained release can be achieved in albumin-based 5-FU formulations.

The distinct biological responses observed for BSA_STR and BSA_5-FU may result from several factors, including differences in drug loading, drug–carrier interactions, intracellular processing, and the intrinsic mechanisms of action of STR and 5-FU. In addition, the different physicochemical properties of the two formulations, particularly their hydrodynamic size, may influence cellular internalization. Particle size can affect both the efficiency and mechanism of nanoparticle uptake, although these processes are also strongly dependent on surface properties, NPs composition, and cell type [61]. Therefore, differences in cellular uptake may contribute to the distinct biological responses observed. However, dedicated uptake and trafficking studies would be required to determine whether BSA_STR and BSA_5-FU follow different internalization pathways.

The data from the present study demonstrate biological activity of the 5-FU-loaded BSA NPs (Table 3), supporting their potential application as a drug delivery system. However, the contribution of drug release to the observed effects cannot be established without direct release studies. Considering the time-dependent effects of 5-FU, future studies should include longer exposure periods and additional PCa cell models, such as PC3, which represents a more aggressive disease phenotype, as well as investigate cellular uptake and intracellular trafficking and explore the co-encapsulation of STR and 5-FU or other therapeutic compounds.

## 4. Conclusions

This study successfully optimized the synthesis of BSA NPs using genipin as a non-toxic crosslinker, establishing a safer and efficient route for the preparation of protein-based nanocarriers. The optimal conditions (24 h reaction time, room temperature, and a 10:1 BSA:genipin ratio) yielded stable, monodisperse, and spherical NPs suitable for drug loading. Under these conditions, encapsulation was highly effective, with STR-loaded BSA NPs reaching an encapsulation efficiency of 82% and 5-FU-loaded NPs achieving 40%, showing the strong loading capacity of this system for pharmacologically distinct drugs. Drug loading also induced marked, drug-dependent changes in hydrodynamic size, highlighting the impact of cargo on nanoparticle properties.

In vitro cytotoxicity studies showed that BSA NPs modulated the biological response to STR and 5-FU in normal (PNT-2) and prostate cancer (22Rv1) cell lines, attenuating the immediate cytotoxic effects of free STR and altering the timing and magnitude of the response to 5-FU. These findings support genipin-based BSA NPs as a promising bio-based nanoplatform for the encapsulation and delivery of therapeutic compounds, with the potential to modulate drug-associated cytotoxicity and improve therapeutic responses in prostate cancer applications. Given the exploratory nature of this work, further studies directly characterizing the release profiles of STR and 5-FU from BSA NPs under physiologically relevant conditions, as well as their cellular uptake and intracellular trafficking, together with in vivo evaluation, will be essential to fully establish the therapeutic potential of this system.

## Figures and Tables

**Table 1:** Parameters tested for the synthesis of BSA NPs, along with their respective hydrodynamic diameter, polydispersity index (PDI), and zeta potential. Results are presented as mean ± SD.

Parameter ^1^		Z-Average (nm)	PDI	Zeta Potential (mV)
Time of reaction (h)	24	404 ± 9	0.151	−11.0 ± 0.1
	72	158 ± 1	1.000	4.4 ± 0.2
Temperature of reaction (°C)	Room temperature	292 ± 5	0.283	−13.5 ± 0.4
	37	>1000 ± 662	0.218	−4.1 ± 0.3
BSA:genipin ratio (*w*/*w*)	10:4	427 ± 4	0.219	−27.4 ± 0.7
	10:1	296 ± 4	0.183	−21.2 ± 0.5
	20:1	258 ± 7	0.156	−20.6 ± 0.9
	40:1	349 ± 3	0.330	−21.4 ± 0.6

^1^ For the reaction time and temperature parameters, a BSA:genipin ratio of 10:1 was used.

**Table 2:** Characteristics of NPs synthesized at room temperature for 24 h and a 10:1 ratio of BSA:genipin, containing encapsulated STR and 5-FU: hydrodynamic size, polydispersity index (PDI), zeta potential, size determined by STEM, and EE%. Results are presented as mean ± standard deviation.

Nanoparticles	Hydrodynamic Size (nm)	PDI	Zeta Potential (mV)	Size (STEM) (nm)	EE (%)
BSA NPs	296 ± 50	0.183	−21.2 ± 0.5	237	n.a.
BSA_STR	422 ± 7	0.206	−27.6 ± 0.2	242	82
BSA_5-FU	217 ± 70	0.147	−20.1 ± 0.3	177	40

n.a., not applicable, as unloaded BSA NPs do not contain an encapsulated active compound.

**Table 3:** Half-maximal inhibitory concentration (IC_50_) values of free drugs, encapsulated drugs, and empty BSA NPs for each cell line.

Samples	IC_50_ (mg/L)			
	PNT-2		22Rv1	
	48 h	72 h	48 h	72 h
STR	5.13	4.63	3.79	4.91
5-FU	*	9.71	3.43	1.17
BSA NPs	205.43	134.45	648.21	590.11
BSA_STR (STR)	1911.76	1652.37	1370.67	4142.35
BSA_5-FU (5-FU)	*	*	350.72	*

* IC_50_ not determined (50% inhibition was not reached).

## Data Availability

The data presented in this study are available on request from the corresponding author.

## Supplementary Materials

The following supporting information can be downloaded at https://www.mdpi.com/article/10.3390/nano16171086/s1, Figure S1: FTIR spectra of native BSA, genipin, and BSA NPs.

## Abbreviations

The following abbreviations are used in this manuscript:

5-FU	5-Fluorouracil
ANOVA	Analysis of variance
BSA	Bovine Serum Albumin
BSA_5-FU	Bovine Serum Albumin encapsulating 5-FU
BSA_STR	Bovine Serum Albumin encapsulating STR
CRC	Colorectal Cancer
DAD	Diode Array Detection
DLS	Dynamic Light Scattering
DMSO	Dimethyl Sulfoxide
EE%	Encapsulation Efficiency
ELS	Electrophoretic Light Scattering
FBS	Fetal Bovine Serum
FEG	Field Emission Gun
I.D.	Internal Diameter
IC	Inhibitory Concentration
HSA	Human Serum Albumin
MTT	3-[4,5-Dimethylthiazol-2-yl]-2,5-diphenyltetrazolium bromide
NPs	Nanoparticles
PBS	Phosphate-Buffered Saline
PCa	Prostate Cancer
PDI	Polydispersity Index
RPMI	Roswell Park Memorial Institute
SD	Standard deviation
SEM	Scanning Electron Microscopy
STEM	Scanning Transmission Electron Microscopy
STR	Sertraline
UHPLC	Ultra-high-performance liquid chromatography
UV	Ultraviolet
