# Supplementary material for: Bovine Serum Albumin Nanoparticles Prepared with Genipin: Size Modulation, Encapsulation, and In Vitro Response
Source: Nanomaterials (Basel). 2026 Aug 31;16(17):1086. doi: 10.3390/nano16171086 (PMC13567307; doi:10.3390/nano16171086)
Supplement: Supplementary file 1 [file nanomaterials-16-01086-s001.zip › nanomaterials-4502660-Supplementary Material.pdf]

Supplementary Material

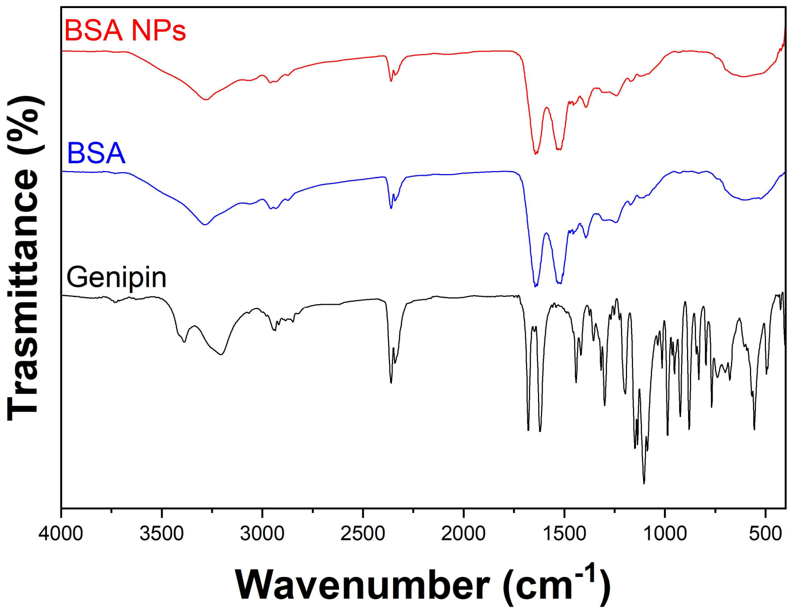

Figure S1: FTIR spectra of native BSA, genipin, and BSA NPs.
